# Association between preoperative serum albumin and prognosis in patients with adrenocortical carcinoma after primary resection: a retrospective study

**DOI:** 10.1186/s12885-021-08689-5

**Published:** 2021-08-26

**Authors:** Fuxun Zhang, Zhihong Liu, Jiayu Liang, Shengzhuo Liu, Kan Wu, Fan Zhang, Chuan Zhou, Yiping Lu, Yuchun Zhu, Xianding Wang

**Affiliations:** Department of Urology, Institute of Urology, West China Hospital, Sichuan University, Chengdu, Sichuan China

**Keywords:** Adrenocortical carcinoma, Prognosis, Resection, Albumin, Nutrition

## Abstract

**Background:**

Adrenocortical carcinoma (ACC) is a rare and aggressive malignancy with a poor prognosis. Given the limited treatment options, prognostic assessment of ACC is increasingly crucial. In this study, we aim to assess the correlation between preoperative serum albumin and prognosis in patients with ACC after primary resection.

**Methods:**

We retrospectively collected and reviewed medical information about 71 ACC patients who underwent primary resection. Survival analysis was performed by Kaplan–Meier analysis with log-rank test or Breslow test. Receiver operating characteristic (ROC) curve and Jordan index was generated to explore optimal cut-off value of albumin. Univariate and multivariate analysis was conducted using Cox’s hazards model. Statistical significance was defined as *P* < 0.05.

**Results:**

Among included patients, 33 patients (46.5%) relapsed at the end of follow-up, while 39 patients (54.9%) died. The median overall survival (OS) of included patients was 17 (range 1–104) months, and median recurrence-free survival (RFS) was 10 (range 0–104) months. In univariate analysis, the albumin was significantly associated with OS (HR:0.491, 95% CI: 0.260–0.930, *P* = 0.029) and RFS (HR: 0.383, 95% CI: 0.192–0.766, *P* = 0.007). In multivariate analysis, serum albumin as an independent prognostic factor of OS was confirmed (HR: 0.351, 95% CI: 0.126–0.982, *P* = 0.046).

**Conclusions:**

Preoperative albumin might be a significant prognostic factor for ACC patients after primary resection. This result may be useful for risk stratification and management of this rare malignancy.

**Supplementary Information:**

The online version contains supplementary material available at 10.1186/s12885-021-08689-5.

## Background

Adrenocortical carcinoma (ACC) is a rare and aggressive malignancy with an overall incidence of 1–2 cases/million per year [1]. Due to mild symptoms, early diagnosis and treatment of ACC are sometimes difficult to reach [2]. Even worse, the rarity of this disease often makes clinical trials and therapeutic options challenging. Meanwhile, radical resection, the most important treatment for ACC, might be impractical for advanced tumors that are common within ACC patients [3]. Moreover, several adjuvant regimens, including mitotane and chemo-radiotherapy, have not previously been evaluated in large randomized trials [4, 5]. Given the heterogeneity and complexity, the management of the ACC continues to be a mission of clinicians worldwide, and the prognostic assessment utilizing markers from routine work-up is increasingly crucial [6].

Malnutrition is correlated with many post-operative complications and adverse outcomes in several cancers [7]. Meanwhile, multiple nutritional markers such as anthropometric measurements, haemoglobin, transferrin, total protein and serum albumin have been recognized as prognostic indicators for various disease [8]. In all, as an objective, quantifiable and potentially reversible parameter, serum albumin level has been widely used to evaluate the nutritional status and prognosis [9].

Albumin is a major protein synthesized by the liver, and makes up primary serum proteins with globulins [10]. In clinical practices, hypoalbuminemia is meant by the concentration of serum albumin lower than 35 g/L, and is mainly associated with malnutrition. Actually, patients with neoplastic diseases are more vulnerable to the hypoalbuminemia due to decreased albumin production from liver and increased losses in ascites or hydrothorax [11]. Meanwhile, previous studies have indicated that hypoalbuminemia was an indicator of systemic inflammatory response associated with deteriorated performance status or tumor progression [12].

Thus, serum albumin maybe an important prognostic predictor for ACC. Here, we aim to investigate the association between preoperative serum albumin and prognosis in patients with ACC after primary resection.

## Methods

### Patients and study protocol

This is a clinician-initiated and retrospective study involved no commercial entity. Data of 90 ACC patients treated from 2009 to 2019 in the West China Hospital were retrieved (Supplement Fig. 1). The main clinicopathological parameters included gender, age, preoperative comorbidities, modus operandi, Ki67 index, preoperative serum albumin level, preoperative globulin and hemoglobin, tumor diameter, tumor hormonal function and tumor stage. Tumor stage was classified by independent radiologists in the same department basing on the American Joint Committee on Cancer (AJCC) 7th edition. The patients included in this study must have to meet the following criteria: Pathological diagnosis of ACC, no previous treatment for any other tumor, available perioperative medical records and laboratory data.

The primary outcome was overall survival (OS) calculated as the time from first surgery to death caused by any reason or last follow-up, as well as the secondary outcome was recurrence-free survival (RFS) defined as the time from first surgery to recurrence or last follow-up. Recurrence was defined as tumor lesion within the radiation field during the follow-up. Besides, follow-up was determined as the time from the primary resection to death or last contact with the patient.

### Statistical analysis

All statistical analyses were performed by using SPSS 23.0 software. Median and ranges or mean value and standard deviation were computed for continuous data. Proportions or rates were calculated for categorical data. The Kolmogorov-Smirnov test was used to identify the distribution of variables. Meanwhile, relevant variables were categorized using appropriate cut-off values to indicate the distribution. The correlation between variables and outcomes were analyzed using Pearson’s or Spearman’s test for continuous variables, and Chi-square test for categorical variables. Survival rates were computed using Kaplan–Meier analysis and compared with log-rank test or Breslow test. Cox’s proportional hazards model was used for univariate and multivariate analysis to identify relative risk and independent prognostic factors. Prognostic role of serum albumin level was analysed as dichotomous variable. Receiver operating characteristic (ROC) curve and Jordan index was generated to explore the optimal cut-off values for dichotomous serum albumin. All *P* values were two-sided and statistical significance was defined as *P* < 0.05.

## Results

### Patients and prognostic factors

After exclusion of 19 patients who did not match with the inclusion criteria, 71 patients were included, and their medical information was collected and reviewed. There were 30 males and 41 female patients, and the median age was 44 (range 2–79) years. Among them, 9 (12.7%) patients and 37 (52.1%) patients were diagnosed at stage I and II, whilst 20 patients (28.2%) and 5 (7.0%) patients at stage III and IV (Tables 1 and 2). At the end of the follow-up period, 33 patients suffered tumor recurrence (46.5%), while 39 patients (54.9%) died. The median OS of included patients was 17 (range 1–104) months and median RFS was 10 (range 0–104) months. The distribution of relevant variables and their association with outcomes was showed in Table 2. Among them, gender, diameter of the tumor, post-recurrence adjuvant treatment, serum albumin and hemoglobin showed the association with outcomes (Table 2).

**Table 1:** Baseline characteristics of included patients

Characteristics	All patients (***N*** = 71)
Male gender [n (%)]	30 (42.3)
Age (years)[median(range)]	44 (2–79)
Hormonal secretion [n (%)]	30 (42.3)
Comorbidities [n (%)]	28 (39.4)
Diameter of tumor (cm)[median(range)]	8.8 (2.3–27.0)
Modus operandi-Laparoscopy [n (%)]	23 (32.4)
Post-recurrence adjuvant treatment [n (%)]	17 (23.9)
Recurrence [n (%)]	33 (46.5)
Death [n(%)]	39 (54.9)
Ki67 index (%)[median(range)]	10 (0–90)
Albumin (g/L)(Mean ± deviation)	40.6 ± 5.1
Globulin (g/L)(Mean ± deviation)	27.6 ± 5.3
Hemoglobin (g/L)(Mean ± deviation)	130.2 ± 21.4

Bold figures indicate statistical significance at *P*<0.05

**Table 2:** The distribution of relevant variables and their association with outcomes

Variables	All patients [n (%)]	Recurrence		Death	
		Events [n (%)]	***P***	Events [n (%)]	***P***
Gender [n (%)]					
Male	30 (42.3)	12 (16.9)	0.349	12 (16.9)	**0.031**
Female	41 (57.7)	21 (29.6)		27 (38.0)	
Age (years)					
≤ 45	38 (53.5)	20 (28.2)	0.265	23 (32.4)	0.309
> 45	33 (46.5)	13 (18.3)		16 (22.5)	
Hormonal secretion [n (%)]					
No	41 (57.7)	19 (26.8)	0.978	25 (35.2)	0.231
Yes	30 (42.3)	14 (19.7)		14 (19.7)	
Comorbidities [n (%)]					
No	43 (60.6)	19 (26.8)	0.631	25 (35.2)	0.501
Yes	28 (39.4)	14 (19.7)		14 (19.7)	
Diameter of tumor (cm)					
≤ 5	16 (22.5)	8 (11.3)	0.704	5 (7)	**0.013**
5–8	24 (33.8)	13 (18.3)		18 (25.4)	
8–10	8 (11.3)	3 (4.2)		2 (2.8)	
> 10	23 (32.4)	9 (12.7)		14 (19.7)	
Tumor stage[n (%)]					
I	9 (12.7)	4 (5.6)	0.977	3 (4.2)	0.389
II	37 (52.1)	17 (23.9)		21 (29.6)	
III	20 (28.2)	10 (14.1)		11 (15.5)	
IV	5 (7.0)	2 (2.8)		4 (5.6)	
Modus operandi [n (%)]					
Laparoscopy	23 (32.4)	13 (18.3)	0.240	10 (14.1)	0.179
Open	48 (67.6)	20 (28.2)		29 (40.8)	
Post-recurrence adjuvant treatment [n (%)]					
No	54 (76.1)	16 (22.6)	**< 0.001**	26 (36.6)	**0.041**
Yes	17 (23.9)	17 (23.9)		13 (18.3)	
Ki67 index (%)					
≤ 5	22 (31.0)	6 (8.5)	0.145	12 (17.0)	0.155
5–10	28 (39.4)	16 (22.5)		14 (19.7)	
10–20	9 (12.7)	6 (8.5)		4 (5.6)	
> 20	12 (16.9)	5 (7.0)		9 (12.7)	
Albumin (g/L)					
≤ 40	35 (49.3)	21 (29.6)	**0.024**	25 (35.2)	**0.006**
> 40	36 (50.7)	12 (16.9)		14 (19.7)	
Globulin (g/L)					
≤ 25	23 (32.4)	11 (15.5)	0.875	15 (21.1)	0.228
> 25	48 (67.6)	22 (31.0)		24 (33.8)	
Hemoglobin (g/L)					
≤ 130	33 (46.5)	20 (28.2)	**0.026**	21 (29.6)	0.169
> 130	38 (53.5)	13 (18.3)		18 (25.4)	

Bold figures indicate statistical significance at *P*<0.05

### Univariate and multivariate prognostic analysis

**Fig. 1:**
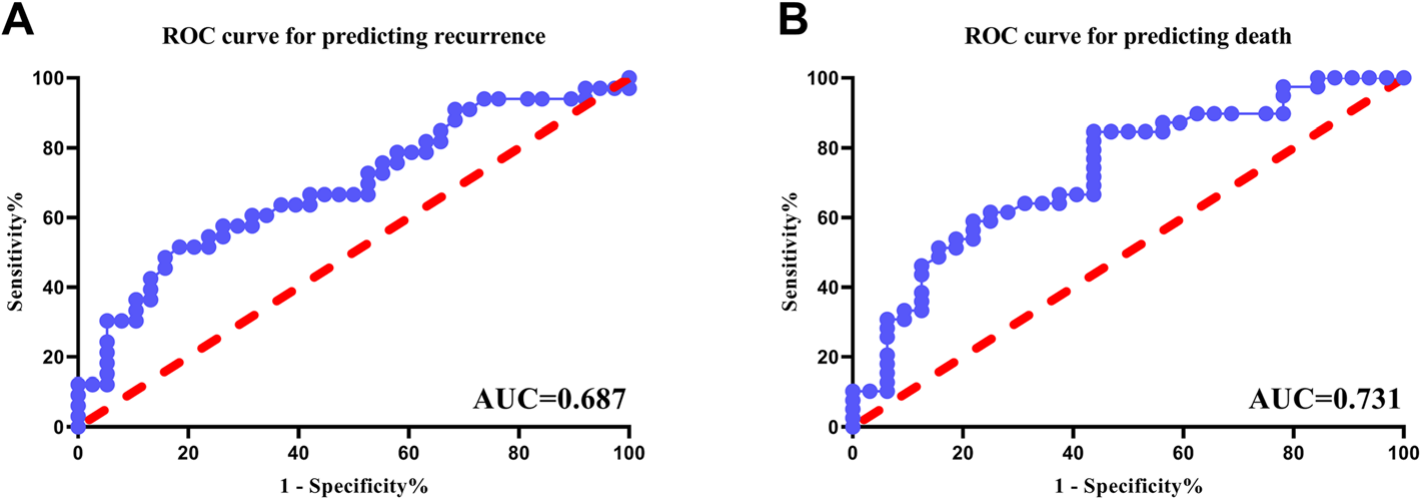
ROC curves were generated to calculate the optimal cut-off values of serum albumin for RFS and OS. Abbreviations: ROC Receiver operating characteristic, RFS recurrence-free survival, OS overall survival

**Fig. 2:**
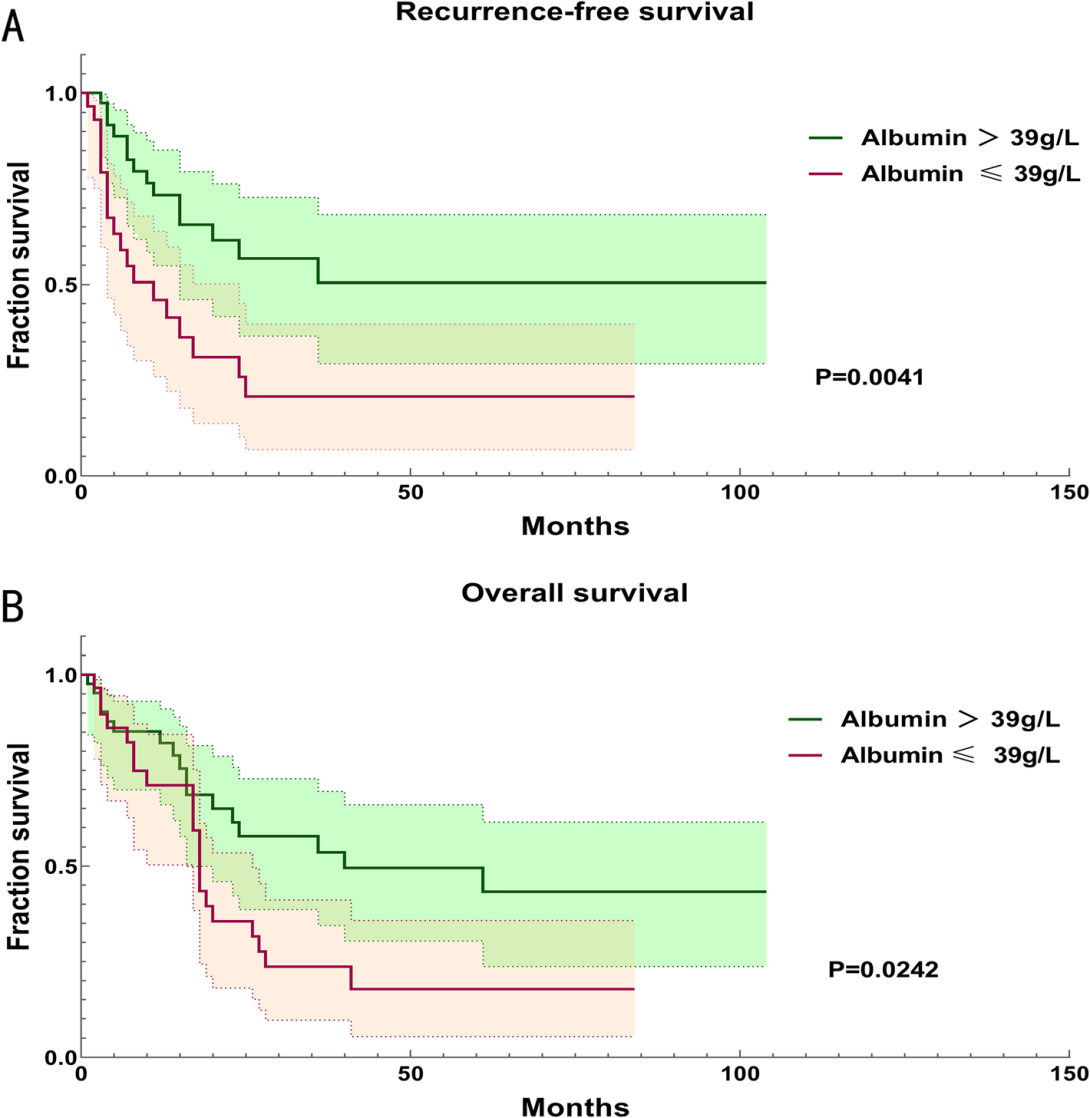
Kaplan-Meier curves of RFS and OS for patients with different serum albumin levels. Shadow areas indicated the 95% CI of each curve. Abbreviations: RFS recurrence-free survival, OS overall survival, CI confidence interval

ROC curve and Jordan index generated to evaluate the optimal cutoff value (Fig. 1). In this study, ROC curve with AUC of 0.687 for predicting recurrence, and 0.731 for death was generated, and the optimal cutoff value of albumin at lower than or equal to 39 g/L was confirmed (Fig. 1). In all, there were 29 patients with albumin≤39 g/Land 42 patients with albumin>39 g/L. The median OS of patients with low (albumin≤39 g/L) and high (albumin>39 g/L) serum albumin level were 18 (range 2–84) months and 16 (range 1–104) months respectively. Meanwhile, the median RFS of each group were 6 (range 0–84) months and 13 (range1–104) respectively. Kaplan–Meier curves showed the significant difference in OS and RFS between low and high serum albumin groups (*P* = 0.0242 and *P* = 0.0041, respectively) (Fig. 2). In univariate analysis, the albumin significantly associated with OS (HR: 0.491, 95% CI: 0.260–0.930, *P* = 0.029) and RFS (HR: 0.383, 95% CI: 0.192–0.766, *P* = 0.007). In multivariate analysis, serum albumin as an independent prognostic factor of OS was confirmed (HR: 0.351, 95% CI: 0.126–0.982, *P* = 0.046). Meanwhile, the present results suggested that albumin might be an independent predictor of RFS (HR: 0.423, 95% CI: 0.176–1.018, *P* = 0.055) (Showed in Tables 3 and 4). All these results indicated that patients with higher preoperative albumin had superior OS and PFS, and preoperative albumin level is related to clinical outcomes. In this section, we also noted that tumor stage (I vs II/III/IV) was significantly associated with OS basing on univariate analysis (HR: 2.533, 95%CI: 1.107–5.793, *P* = 0.037). Meanwhile, Ki67 index demonstrated prognostic significance for OS in multivariate analysis (HR: 1.043, 95% CI: 1.015–1.072, *P* = 0.046), and hemoglobin showed potential prognostic relevance for RFS in univariate analysis (HR: 0.974, 95% CI: 0.955–0.992, *P* = 0.006) (Tables 3 and 4).

**Table 3:** Univariate analysis of relevant variables for OS and RFS

Variables	RFS			OS		
	HR	95%CI 1(ref)	***P***-value	HR	95%CI 1(ref)	***P***-value
Gender						
Male	1.462	0.717–2.981	0.296	1.721	0.869–3.410	0.119
Female						
Age (years)	0.995	0.974–1.016	0.638	0.997	0.977–1.019	0.813
Hormonal secretion						
No	1.059	0.530–2.118	0.870	0.687	0.357–1.324	0.262
Yes						
Comorbidities						
No	1.082	0.542–2.162	0.823	0.759	0.394–1.461	0.409
Yes						
Diameter of tumor (cm)						
≤ 8.8	1.027	0.517–2.039	0.940	1.345	0.717–2.525	0.356
> 8.8						
Tumor stage						
I	1.257	0.441–3.580	0.669	2.533	1.107–5.793	**0.037**
II/III/IV						
Modus operandi						
Laparoscopy	0.925	0.459–1.867	0.829	1.849	0.899–3.803	0.095
Open						
Post-recurrence adjuvant treatment						
No				1.118	0.572–2.185	0.745
Yes						
Recurrence						
No				1.769	0.922–3.394	0.086
Yes						
Albumin (g/L)						
≤ 39	0.383	0.192–0.766	**0.007**	0.491	0.260–0.930	**0.029**
> 39						
Ki67 index (%)	1.024	0.999–1.050	0.059	1.034	1.011–1.057	**0.004**
Globulin (g/L)	0.989	0.929–1.054	0.741	0.994	0.940–1.052	0.846
Hemoglobin (g/L)	0.974	0.955–0.992	**0.006**	0.992	0.978–1.006	0.257

Bold figures indicate statistical significance at *P*<0.05

*Abbreviations*: *OS* overall survival, *RFS* recurrence-free survival, *HR* hazard ratio, *CI* confidence interval, *1 (ref)* 1 degree of freedom

**Table 4:** Multivariate analysis of relevant variables for OS and RFS

Variables	RFS			OS		
	HR	95%CI 1(ref)	***P***-value	HR	95%CI 1(ref)	***P***-value
Gender	1.272	0.545–2.970	0.578	2.210	0.792–6.163	0.130
Age	0.991	0.965–1.017	0.493	0.987	0.958–1.018	0.411
Hormonal secretion	0.883	0.385–2.024	0.768	0.770	0.325–1.823	0.551
Comorbidities	0.951	0.413–2.192	0.906	0.561	0.228–1.384	0.210
Diameter of tumor	0.938	0.375–2.344	0.891	1.333	0.564–3.154	0.512
Tumor stage	1.079	0.408–2.858	0.878	0.664	0.271–1.626	0.370
Modus operandi	0.773	0.279–2.143	0.621	1.664	0.575–4.820	0.348
Post-recurrence adjuvant treatment				0.540	0.186–1.567	0.257
Recurrence				2.437	0.910–6.526	0.076
Albumin	0.423	0.176–1.018	0.055	0.351	0.126–0.982	**0.046**
Ki67 index	1.024	0.998–1.052	0.074	1.043	1.015–1.072	**0.003**
Globulin	0.971	0.902–1.045	0.430	0.999	0.919–1.087	0.987
Hemoglobin	0.985	0.962–1.008	0.196	1.013	0.993–1.034	0.196

Bold figures indicate statistical significance at *P*<0.05

*Abbreviations*: *OS* overall survival, *RFS* recurrence-free survival, *HR* hazard ratio, *CI* confidence interval, *1 (ref)* 1 degree of freedom

## Discussion

ACC is a rare and aggressive malignancy lacking effective treatment [13]. Although this heterogeneous disease is always kept in focus, the management and prognostic assessment of ACC remains a challenge for clinicians worldwide. Owing to the absence of randomized controlled trials, radical resection is still the only curative treatment, while no chemo-radiotherapy or other adjuvant regimens had yet revealed long-term benefits for patients with ACC [6]. Besides, adjuvant therapies aiming to decrease recurrence are comprised of outdated regimens with substantial side effects and unwanted clinical efficacy [14]. In order to assess the prognosis for ACC patients, many prognostic studies have been performed, and several predictors developed [15]. Among them, resection status, tumor stage and grade are regarded as the most informative predictors of ACC [6]. However, current prognostic markers are restricted by intrinsic limitations which might reduce their utility in ACC [2, 16].

The tumor stage has been considered as the cornerstone of the prognostic stratification for ACC patients [17]. However, the T-stage classification of the ACC is still being amended, and the standardization of imaging and pathological classification requires further improvement [2]. In this study, we noted that the tumor stage is associated with OS, and patients in stage I might have longer survival (Table 3). However, perhaps because of small sample size, the tumor stage did not demonstrate the dependent significance in the multivariate analysis. Additionally, there were only 9 (12.7%) patients diagnosed in stage I, emphasizing the aggressiveness of the ACC and the necessity of early intervention. Surgical margin status was regarded as an important predictor for patients survival, favoring the application of the T-stage classification [18]. However, considering that most ACC are large and locally advanced, the judgement of margin status often involves subjective judgment and clinical experience of the surgeon, which might contribute to the bias and unreliability of this marker. Taken together, we hold the opinion that these progression-associated markers may not be the best predictors for ACC. Consequently, exploiting a common parameter rather than searching for biomarkers in vitro may be more valuable for this rare malignancy to provide rapid application in prognostic evaluation and patients stratification.

As a common biochemical marker used for assessing nutritional performance, serum albumin level has demonstrated prognostic value in several benign or malignant diseases [19, 20]. Meanwhile, it is recognized that serum albumin could provide a prognostic value with other markers [21]. In line with previous studies, our study indicates that the preoperative albumin level is a potential prognostic factor for ACC. Specifically, patients with higher preoperative albumin may have a better prognosis, by comparison, those with lower preoperative albumin suffered remarkable reduction in RFS and OS (Fig. 2). Unexpectedly, serum albumin did not demonstrate statistical prognostic significance for RFS in Cox multivariate analysis (HR:0.423, 95%CI: 0.176–1.018, *P* = 0.055) (Table 4). However, it has shown a marginal significance for RFS, and the Kaplan-Meier curves of RFS were statistically different (*P* = 0.0041) (Fig. 2A), indicating that preoperative albumin might be a prognostic marker for RFS.

In this study, we also noticed that the cutoff value of albumin (39 g/L) is greater than previous studies of other cancers [7, 22]. For this discrepancy, we hold the opinion that the number of patients with adrenal incidentaloma who underwent early radical surgery is increasing in recent years. Although incidentaloma would be replaced by ACC according to post-operative pathological diagnosis, these patients with adrenal incidentaloma possessed excellent nutritional performance, and may influence the results. Additionally, ACC patients who were incidentally detected might have a better survival.

The complex mechanisms between serum albumin and prognosis in malignancy remain to be investigated. Maybe malnutrition characterized by hypoalbuminemia tends to incur more postoperative complications, and subsequently increase the mortality and morbidity hazard in some degree. On the other hand, the serum albumin with short half-life is prone to be influenced by several conditions such as diabetes mellitus, hepatic insufficiency, chronic kidney disease and hypercortisolism, which may in the end have adverse effects on the prognosis of ACC. Particularly, it is reported that in ACC patients cortisol-secretion significantly increase the risk of poor outcomes, including recurrence and death, and should be deemed as a negative prognostic factor [23]. Moreover, many cytokines from systemic inflammatory response related to tumor progression may inhibit the albumin synthesis, and accelerate the albumin loss to interstitial space [12, 24]. In fact, there are various ways of assessing malnutrition, such as body mass index (BMI), weight loss, arm circumference and food-intake [25]. However, these assessments were performed by prospective studies characterized by subjective and qualitative properties, and might be impracticable for this retrospective study [26].

Radical resection is still the only choice to cure ACC so far [14]. Although the perioperative management and surgical techniques are being refined recently, surgery remains an invasive approach with potential risk of complications, sometimes seriously. Thus, risk stratification is essential for cancer patients, especially for those with rare malignancy. Although various predictors have been reported, preoperative albumin appears to be a significant prognostic factor for long-term survival in this study. Moreover, hypoalbuminemia as a representative of malnutrition could be evaluated conveniently. Therefore, preoperative serum albumin might be an important prognostic marker for ACC, and adequate nutrient supplementation prior to resection might improve the survival of ACC patients.

It is reported that survival is associated with age [18]. In this study, there was no statistical correlation between age and outcomes, which might be caused by small sample size. However, on the other hand, this result might indicate that the high malignancy of ACC was independent of age. Moreover, hemoglobin carried significance related to RFS in univariate analysis. Actually, lower hemoglobin level or anemia is prevalent in cancer patients, and is caused by various factors [27]. Therefore, lower hemoglobin in the current study might simply result from malnutrition, and might be irrelevant to RFS.

Generally, Ki67 index as an important proliferation biomarker has been incorporated into the routine of diagnosis and prognostic assessment of ACC [14]. We also found that Ki67 index reached the statistical significance of OS, and demonstrated the marginal correlation with RFS in multivariate analysis, which is in line with our previous article [28]. Although the interpretation of Ki67 index on the basis of pathological specimens is susceptible to inter-laboratory/observer variation, and still need to be improved, we hold the opinion that Ki67 index is a reliable marker in the prognostic assessment of ACC.

This study has several limitations. Given the heterogeneity of ACC, patients with lower albumin maybe suffered more aggressive tumors than others, and thus their conditions may deteriorate rapidly. Moreover, due to the retrospective design and the long time span of data, some variables related to nutritional status, especially the BMI, are not available, which have different degrees of bias on the results. Additionally, statistical power in present study was compromised by the small sample size since the rarity of the ACC. For example, age identified as a predictor of survival in previous reports did not demonstrate prognostic value in our cohort, which might be attributed to small sample size. Taken together, these factors influenced the prognostic significance of albumin in ACC, and weaken the power of this study. Thus, further explore is required to validate our findings. Meanwhile, retrospective investigations should be continued to collect data and provide more evidence for prognostic assessment of this rare malignancy.

## Conclusions

In this study, clinical characteristics of ACC were analysed. Among them, preoperative serum albumin might be a significant prognostic factor for ACC patients after primary resection. This result may be useful for risk stratification and management of ACC. Additionally, our results also suggested that nutritional repletion prior to surgery may improve the long-term outcomes of ACC patients.

## Supplementary Information

**Additional file 1: Supplementary Fig. 1** Flow chart. Abbreviations: RFS recurrence-free survival, OS overall survival.

## Data Availability

The datasets analyzed during the current study are available from the corresponding author on reasonable request.

## Abbreviations

ACC: Adrenocortical carcinoma
ROC: Receiver operating characteristic
OS: Overall survival
RFS: Recurrence-free survival
HR: Hazard ratio
CI: Confidence
BMI: Body mass index
