# Supplementary figures and images for: Association between preoperative serum albumin and prognosis in patients with adrenocortical carcinoma after primary resection: a retrospective study
Source: BMC Cancer. 2021 Aug 26;21:961. doi: 10.1186/s12885-021-08689-5 (PMC8393459; doi:10.1186/s12885-021-08689-5)

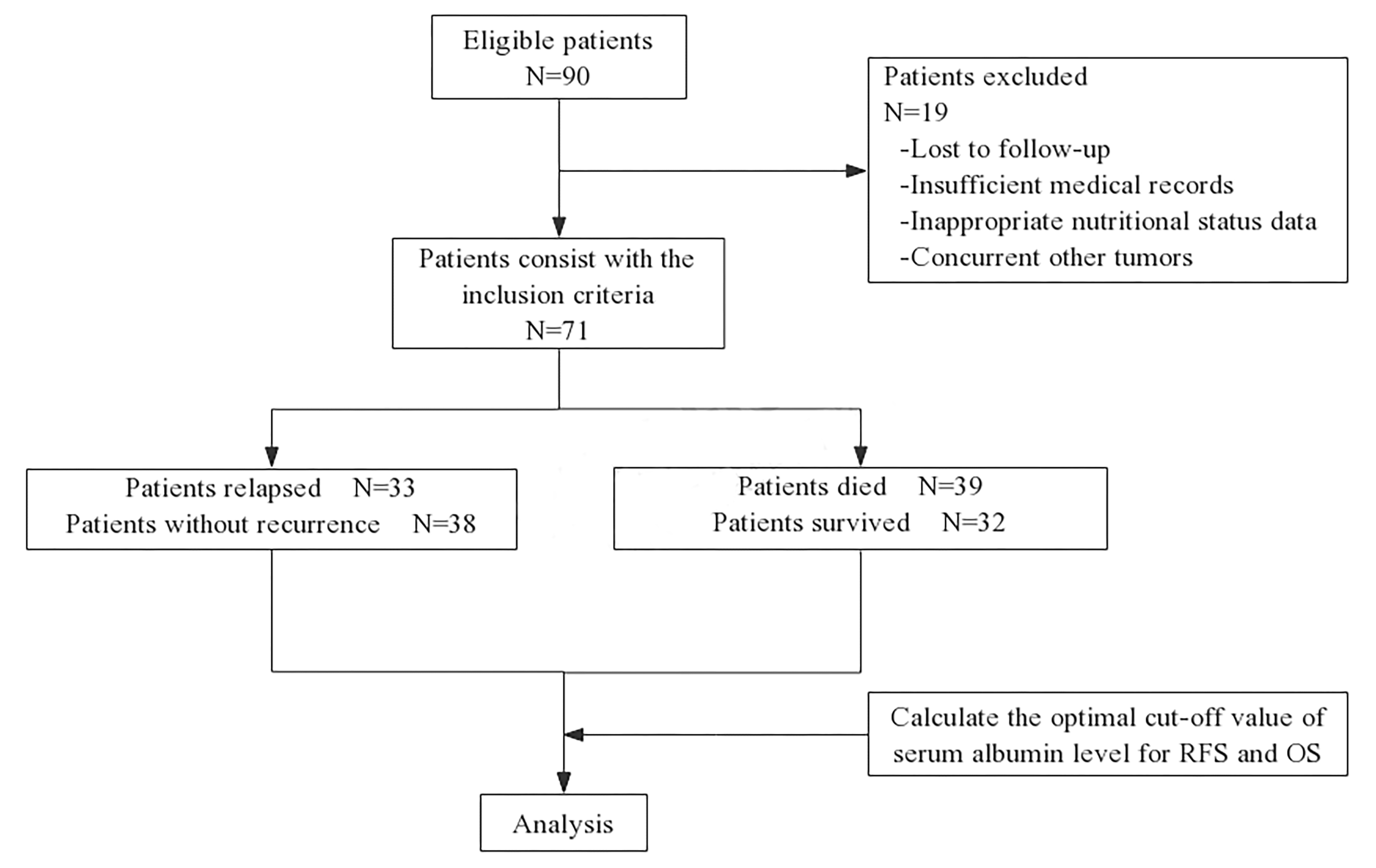

Supplement: Supplementary file 1 — Additional file 1: Supplementary Fig. 1 Flow chart. Abbreviations: RFS recurrence-free survival, OS overall survival. [file 12885_2021_8689_MOESM1_ESM.tif]
